# Clinical and economic outcomes evaluated in Lyme disease: a systematic review

**DOI:** 10.1186/s13071-020-04214-y

**Published:** 2020-07-09

**Authors:** T. Joseph Mattingly, Kalpana Shere-Wolfe

**Affiliations:** 1 University of Maryland School of Pharmacy, Baltimore, Maryland USA; 2 University of Maryland Institute of Human Virology, Baltimore, Maryland USA

**Keywords:** Lyme disease, Value, Outcomes, Cost

## Abstract

**Background:**

The financial implications of Lyme disease (LD) can vary widely for both the health system and the individual patients experiencing the disease. The aim of this review was to summarize published data on clinical and economic outcomes associated with LD.

**Methods:**

A literature review was conducted to identify all studies of LD that incorporate both clinical outcomes and costs. Included studies were described and categorized based on costs consistent with best practices used in economic evaluation.

**Results:**

The most frequent costs identified focused on formal health costs and productivity losses were the most common costs identified outside of the health system. Travel and informal care costs were less frequently reported. Clinical and economic outcomes of LD are primarily studied through economic models or observational analyses and focus on formal health care.

**Conclusions:**

This review provides and overview of existing evidence and recommendations for future economic analyses in LD.
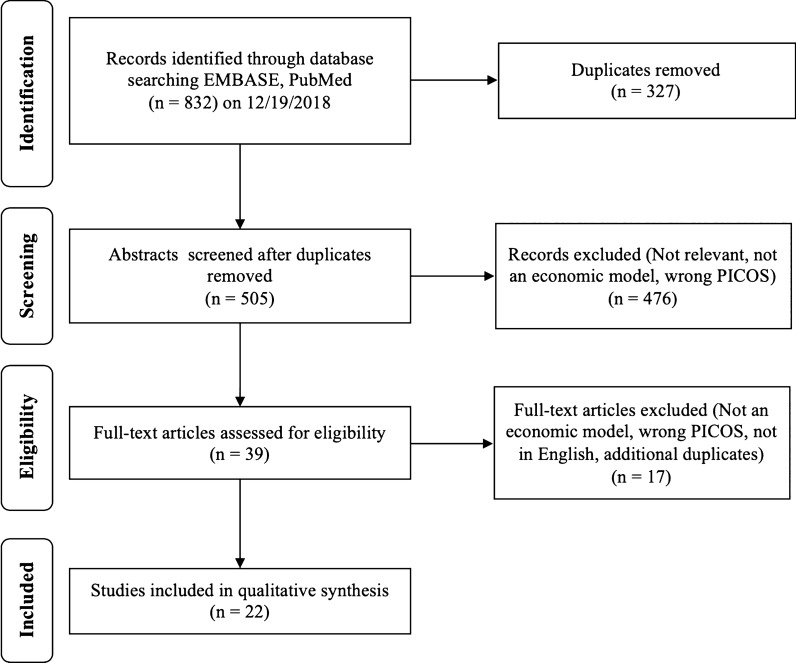

## Background

Exposure to the bacterium *Borrelia burgdorferi* (*sensu lato*), typically transmitted by blacklegged ticks, is the cause of Lyme disease (LD) according to the Centers for Disease Control and Prevention [1]. Clinically, LD is categorized by early and late disease manifestations [2]. Following a tick bite, the clinician may consider multiple options, including antimicrobial prophylaxis if the suspected bite occurred within the previous 72 hours, to prevent progression to early LD [3]. After the diagnosis of early LD has been made, systematic evidence and guideline recommendations support oral beta-lactams (amoxicillin, cefuroxime axetil) and oral tetracyclines (doxycycline) as effective first-line agents based on nine randomized, prospective studies where erythema migrans (an expanding red skin rash) was the disease-defining criterion and the resolution of symptoms the most common outcome [2]. Other early manifestations of disease may include meningitis, carditis, arthritis, or neurological symptoms [4].

While antibiotic treatment of early LD may improve the cutaneous symptoms and prevent other early disease complications, only 10–15% of patients develop a late stage disease, commonly referred to as post-treatment Lyme disease syndrome (PTLDS) or late LD [5]. Despite treatment, some patients will develop PTLDS characterized by nonspecific symptoms such as fatigue, arthralgia and cognitive disturbances. With only a fraction of patients observed in early disease trials progressing on to symptoms synonymous with PTLDS, it is difficult to reach an appropriate sample size to critically evaluate effectiveness between comparators or other sub-group questions linking early disease treatment and PTLDS. PTLDS is frequently characterized by symptoms such as musculoskeletal pain, fatigue and cognitive difficulties which may overlap with other diseases such as fibromyalgia or chronic fatigue syndrome [5]. Early disease presentation and treatment is relatively straightforward, with little controversy among practitioners. For the proportion of patients who go on to experience the PTLDS complications, the experience can be particularly challenging. A qualitative, phenomenological study of 12 PTLDS patients reported four major themes describing patient experiences and perceptions: (i) changing health status and social impact; (ii) doubts about recovery and the future; (iii) contrasting doctor-patient relationships; and (iv) the use of unconventional therapies [6].

When health economists develop studies to estimate costs attributable to a given disease, the methodological approach, perspective of the study and source of data may greatly influence the findings and the impact factor of the journal where the results are published [7–9]. These “cost-of-illness” evaluations help translate the burden of disease into dollars, which may be more impactful in the policy arena as decision-makers debate budget priorities [10]. The variation in methods used in these studies frequently lead to a wide variation of cost estimates for the same disease, limiting the comparability across studies, but provide a necessary first step to account for the economic burden of the disease [11]. In the case of LD, the financial implications of the disease can vary widely for both the health system and the individual patients experiencing the disease. Previous reviews identified both a range in the methods of estimating costs or cost-effectiveness of treatments or vaccinations for LD [12, 13]. On the one hand, the prevalence of LD reduces the overall burden of LD at the country-level, but from the patient perspective the costs can be quite substantial [13]. The aim of this review was to summarize published data on clinical and economic outcomes associated with LD. We also provide recommendations for future cost-effectiveness analyses conducted to evaluate the benefit of LD treatment.

## Methods

Our systematic review adhered to the Preferred Reporting Items for Systematic Reviews and Meta-analyses (PRISMA) statement [14] (Additional file 1: Table S1). Due to the heterogeneous nature of publications that include both clinical outcomes and cost estimates, this review only provided an assessment of the studies without a summary estimate from a meta-analysis. A literature search was conducted using EMBASE and PubMed, including studies published through 19 December 2018. The search terms included (((((“lyme*”[Title/Abstract]) OR “borrelia*”[Title/Abstract]) OR “erythema chronicum migrans*”[Title/Abstract]) OR “erythema migrans*”[Title/Abstract])) AND (((((cost*[Title/Abstract]) OR economic*[Title/Abstract]) OR budget*[Title/Abstract]) OR financ*[Title/Abstract]) OR burden*[Title/Abstract]) and results were limited to peer-reviewed publications in English. Titles and abstracts were screened by multiple reviewers for relevance to infectious diseases where LD was reported. Abstracts were reviewed by two authors for screening, with any inclusion discrepancies being discussed for consensus. Original research full-text manuscripts focused on the LD population were included if both clinical and economic outcomes were reported. Studies focused on clinical efficacy or comparative effectiveness without assessing costs or cost-effectiveness were excluded.

Risk of bias was assessed by considering study rigor, type of analysis used, and population as described in the results. Data extraction included the following variables: authors, article title, journal title, year published, type of study, population, outcomes identified in study, any costs identified in study, study funder, and any evidence of direct patient engagement used throughout the study. The type of study was categorized by the research approach which may include clinical trial, observational research using real-world evidence, economic model or simulation-based economic analysis, mixed-methods surveys and qualitative interviews. A narrative synthesis of the findings was structured around the target population characteristics, type of outcome, and type of costs identified. Costs were categorized based on recommendations of the Second Panel on Cost Effectiveness in Health and Medicine (“Second Panel”) that include formal health sector, informal health sector, non-health sector, and other [15]. Formal health sector costs include items such as the costs of medication, inpatient or outpatient care, laboratory tests, or other specialized care directly related to use or consumption of health services. Informal health sector costs include items such as patient or caregiver time or transportation costs that arise when using health services but are often not covered by health insurance. Non-health sector costs capture items such as productivity losses that fall outside the health sector but are often evaluated for the societal perspective in an economic evaluation.

## Results

**Fig. 1:**
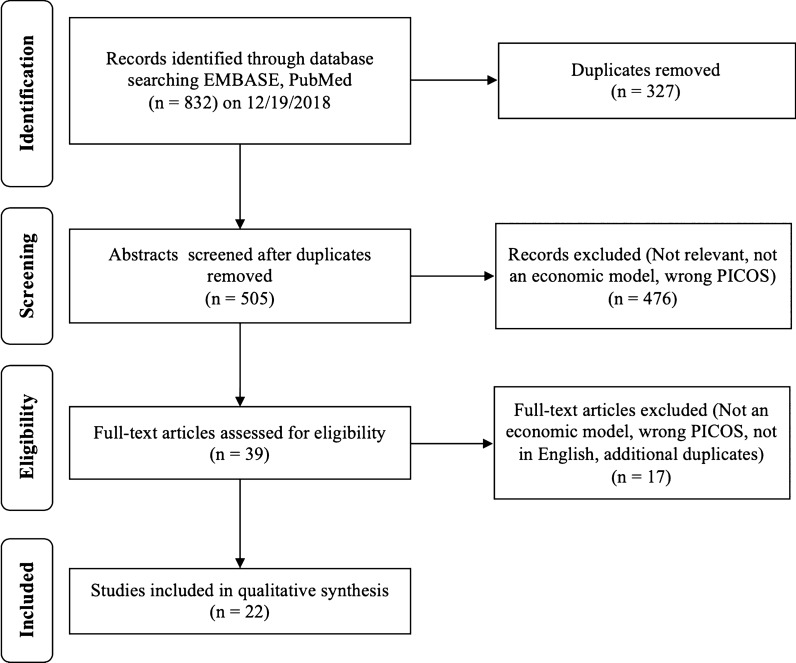
Review flow diagram according to the preferred reporting items for systematic reviews and meta-analyses: the PRISMA statement [14]

A total of 832 abstracts were found during the initial search with 327 duplicates, for a total of 505 articles for screening. An additional 476 articles were removed during abstract screening due to lack of relevance or study type. Reviewer agreement for inclusion during screening was 92.7% (468/505). Disagreements were addressed and a total of 39 articles were included in the full-text review. An additional 15 articles were excluded after full-text review for lack of relevance, study type, not a full manuscript, or inappropriate comparators or outcomes assessed. A total of 24 articles were included for qualitative synthesis and full data extraction table is available in Table 1 [6, 16–36]. The full results of the search strategy are provided in a PRISMA flow chart (Fig. 1) [14].

**Table 1:** Summary of characteristics for included studies

Reference	Title	Study type	Country	Funding	Patient engagement
Ali et al. (2014) [6]	Experiences of patients identifying with chronic Lyme disease in the healthcare system: a qualitative study	Qualitative, in-depth interviews	USA	Government	Yes
Berende et al. (2018) [16]	Cost-effectiveness of longer-term *versus* shorter-term provision of antibiotics in patients with persistent symptoms attributed to Lyme disease	Economic analysis (alongside clinical trial)	Netherlands	Government	No
Boudreau et al. (2018) [27]	Motivations and experiences of Canadians seeking treatment for Lyme disease outside of the conventional Canadian health-care system	Survey; semi-structured; qualitative	Canada	None	Yes
Drew & Hewitt (2006) [30]	A qualitative approach to understanding patients’ diagnosis of Lyme disease	Qualitative, in-depth interviews	USA	Private non-profit	Yes
Eckman et al. (1997) [31]	Cost effectiveness of oral as compared with intravenous antibiotic therapy for patients with early Lyme disease or Lyme arthritis	Economic analysis (model)	USA	Government	No
Fix et al. (1998) [32]	Tick bites and Lyme disease in an endemic setting	Observational (prospective cohort)	USA	Government	No
Gasmi et al. (2017) [33]	Practices of Lyme disease diagnosis and treatment by general practitioners in Quebec, 2008–2015	Observational (retrospective cohort)	Canada	Government	No
Henningsson et al. (2010) [34]	Neuroborreliosis - an epidemiological, clinical and healthcare cost study from an endemic area in the south-east of Sweden	Observational (retrospective cohort)	Sweden	Private non-profit	No
Hsia et al. (2002) [35]	Cost-effectiveness analysis of the Lyme disease vaccine	Economic analysis (model)	USA	Private non-profit	No
Johnson et al. (2011) [36]	Healthcare access and burden of care for patients with Lyme disease: a large United States survey	Survey; quantitative	USA	None	Yes
Johnson et al. (2014) [17]	Severity of chronic Lyme disease compared to other chronic conditions: a quality of life survey	Survey; quantitative	USA	None	Yes
Joss et al. (2003) [18]	Lyme disease - what is the cost for Scotland?	Observational (prospective cohort)	Scotland	Government	No
Lantos et al. (2013) [19]	Empiric antibiotic treatment of erythema migrans-like skin lesions as a function of geography: a clinical and cost effectiveness modeling study	Economic analysis (model)	USA	None	No
Lightfoot Jr et al. (1993) [20]	Empiric parenteral antibiotic treatment of patients with fibromyalgia and fatigue and a positive serologic result for Lyme disease: a cost-effectiveness analysis	Economic analysis (model)	USA	None	No
Lohr et al. (2015) [21]	Epidemiology and cost of hospital care for Lyme borreliosis in Germany: lessons from a health care utilization database analysis	Observational (cost-of-illness)	Germany	Government	No
Maes et al. (1998) [29]	A cost-of-illness study of Lyme disease in the United States	Economic analysis (cost-of-illness)	USA	Private for-profit	No
Magid et al. (1992) [22]	Prevention of Lyme disease after tick bites - a cost-effectiveness analysis	Economic analysis (model)	USA	Government	No
Meltzer et al. (1999) [23]	The cost effectiveness of vaccinating against Lyme disease	Economic analysis (model)	USA	Government	No
Nichol et al. (1998) [24]	Test-treatment strategies for patients suspected of having Lyme disease: a cost-effectiveness analysis	Economic analysis (model)	USA	None	No
Shadick et al. (2001) [25]	The cost-effectiveness of vaccination against Lyme disease	Economic analysis (model)	USA	Government	No
van den Wijngaard et al. (2017) [26]	The cost of Lyme borreliosis	Observational (cost of illness)	Netherlands	Government	Yes
Zhang et al. (2006) [28]	Economic impact of Lyme disease	Observational (case-control)	USA	Government	Yes

Of the articles included for analysis, 68% (15/22) were based in the USA, 9% (2/22) in Canada, 9% (2/22) in the Netherlands and only single studies published for populations in Sweden, Scotland and Germany. The majority (55%, 12/22) of the studies were supported by government funding, with “no funding disclosed” (27%, 6/22) and private funding (18%, 4/22) through either a non-profit foundation or for-profit corporation the other two sources of research funding. In terms of patient engagement, only 32% (7/22) incorporated methods that directly engaged with patients either through in-depth interviews or surveys. No studies listed patients or patient caregivers as members of the research team.

Study methods varied from economic models (45%, 10/22), observational studies using real world evidence (29%, 7/24), mixed-method surveys and qualitative interviews (21%, 5/24). Economic models used simulation methods to estimate costs or cost-effectiveness of different treatments or vaccines. Observational studies included cost-of-illness analyses, prospective cohorts and retrospective cohorts. There were no clinical trials that met the review inclusion and exclusion criteria.

Table 2 summarizes the most frequent clinical outcomes assessed in LD evaluations with clinical manifestations related to rheumatological, neurological, cardiological and dermatological disorders. Items such as “system challenges” that describe the frustration many LD patients report throughout the course of diagnosis and treatment were identified in a few studies. Psychological impact of disease related to items such as stress, anxiety, or depression were only identified in two studies.

**Table 2:** Most frequent outcomes identified in the context of Lyme disease

Outcomes	No. of studies	Brief description
Rheumatologic	7 [21–23, 25, 26, 28, 29]	Including Lyme arthritis, myalgia, or other musculoskeletal manifestations
Neurological	7 [21–23, 25, 26, 28, 29]	Including encephalopathy, peripheral neuropathy, neuroborrelisosis, bell palsy and meningitis
Cardiological	6 [22, 23, 25, 26, 28, 29]	Including Lyme carditis or other defined cardiac sequelae
Dermatological	5 [22, 25, 26, 28, 29]	Including erythema migrans, acrodermatitis and chronica atrophicans
System challenges	5 [6, 20, 27, 30, 36]	Frustration with diagnostic delay, access issues, multiple providers, out-of-network providers and other negative system experiences
Adverse events	4 [19, 20, 25, 35]	Including major and minor events related to antibiotic treatment or vaccination strategies
Quality of life	4 [16, 17, 27, 30]	Including quality-adjusted life-year measures or other specific health related quality of life estimates
Other Lyme sequelae	3 [25, 26, 29]	Including lymphocytoma, ocular manifestations, headache, acute back pain and neck pain
Psychological	2 [6, 30]	Including stress, anxiety, or depression

Table 3 summarizes the most frequent costs with formal health sector costs reported in the majority of studies. Productivity losses due to illness were the most common costs identified outside of the health sector. Health system issues, travel and informal care costs were less frequently identified.

**Table 3:** Most frequent costs identified in the context of Lyme disease

Costs	No. of studies	Brief description	Cost category
Intervention	18 [17, 18, 20–26, 28, 29, 31–35]	Antibiotic treatments or vaccine interventions studied	Formal health sector
Health resource utilization	17 [17, 18, 20–26, 28, 29, 31, 32, 34, 35]	Office visits, hospitalizations, management of adverse events and other direct medical costs pursuant to common Lyme sequelae	Formal health sector
Productivity losses	10 [16–18, 21, 23, 26, 28–30, 36]	Any absenteeism or presenteeism costs due to disease	Non-health sector
Serological testing	9 [18, 22–26, 28, 32, 33]	Diagnostic testing	Formal health sector
Health system issues	5 [6, 20, 28, 30, 36]	Costs experienced with health system navigation including diagnostic delays, unconventional therapies, costs of inconvenience and costs related to concern for false positive tests	Other
Travel expenses	4 [16, 27, 28, 36]	Any travel expenses related to Lyme sequelae	Informal health sector
Informal care costs	2 [26, 28]	Caregiver time costs	Informal health sector

## Discussion

Most studies evaluating both the clinical and economic impact of LD focused on direct medical costs frequently identified as part of the formal health sector perspective in economic evaluations [15]. Non-health sector costs were identified in several studies, but these were limited to productivity losses (absenteeism and presenteeism). Other non-health sector cost categories such as consumption, social services, education, housing, or environment were not identified [15]. In 2018, the International Society of Pharmacoeconomics and Outcomes Research formed a special task force on value in health care that recognized several elements of value not frequently identified in conventional economic evaluations including “reduction in uncertainty, fear of contagion, insurance value, severity of disease, value of hope, real option value, equity, and scientific spillovers” [37]. When considering potential elements of value that may be of interest to LD patients, many of these areas have yet to be explored in a more formal, systematic way. When LD patients were engaged directly through either structured interviews or surveys, uncertainty regarding diagnosis and treatment are common themes and sources of frustration for patients [6, 27, 30, 36].

Studies that capture both direct and indirect costs of LD demonstrate how perspective impacts costs estimates. For example, Lohr et al. [21] estimated that median costs for LD hospitalizations in Germany from 2008 to 2011 were €3917 for adolescents and €2843 for adults, or about 23.7 million Euros annually. They further estimated indirect costs based on productivity and work absences to account for an additional 7.1 million Euros [21]. van den Wijngaard et al. [26] included both direct and indirect costs in their cost-of-illness analysis of LD in the Netherlands to estimate the mean costs were approximately €5700 per patient annually in 2014. Maes et al. [29] estimated direct and indirect costs of LD in the USA to vary greatly between early and late disseminated disease. They reported direct costs in 1996 ranging from 731–3445 USD per year and 2740–8270 USD per year for early and late disseminated disease, respectively [29]. When extending to indirect costs from time or productivity loss, costs ranged from as low as 89–3152 USD, influenced significantly by the severity of disease [29].

Economic evaluation focusing on the direct costs associated with an intervention and cost savings related to formal care are useful for health insurers (public or private) typically responsible for purchasing these services as part of the medical or pharmacy benefits in the patient’s health plan. Direct health care costs are frequently assessed to inform this payer perspective. For example, an observational study by Adrion et al. [38] focusing on PTLDS estimated that 52,795 commercially insured patients experienced an average of 2968 USD higher direct health care costs annually compared to matched controls. Unfortunately, this limited focus may miss significant costs experienced by the patient that also influence decision-making at the patient- and provider-level. Additionally, if the evaluation solely focuses on the impact on the individual patient, any potential spillover effects on the immediate family or caregivers involved [39, 40].

In the case of LD, many patients report other factors that are not typically incorporated into cost-effectiveness analyses. When an LD patient experiences frustration with the health system due to system challenges such as a provider being dismissive of the diagnosis, it is difficult to capture the economic consequences that may result from the negative patient-provider interaction [6]. These patient experience variables are unavailable in cost-of-illness studies using administrative claims data to capture costs while the cost data are often missing from prospective clinical studies where the patient experience may be better documented.

In some cases, researchers focus on broader country-level economic burden or impact of LD on a total population. This may be very useful for determining whether investing in universal vaccinations for a large population would be cost effective or if a more targeted strategy would be preferred [12]. Similar to studies focused on the costs attributed to LD, the approach to costing (direct health costs or including non-health costs) plays a role in the evaluation. However, these studies are quite sensitive to incidence rates, so ensuring accurate diagnoses of new cases is critical to the accuracy of these models [12, 35]. Additionally, public perceptions and trust in vaccine recommendations may also influence one’s willingness-to-pay for a vaccine [41]. Considering communication strategies or efforts to improve these other factors may be important for economic models that focus on vaccines and prevention.

This review was limited by study type and methodological variation within studies identified for inclusion, limiting the ability for the quantitative synthesis of costs for each category. Including studies from all over the world also limits the generalizability to patients within any single country’s health system. For example, costs of copayments and other out-of-pocket expenditures may not be as significant for patients residing in a country with a single-payer system with less coinsurance expectations on the population. Our targeted search strategy focused on abstract and titles for keywords may have limited the number of potential abstracts for review. Additionally, we screened to focus on studies that included both clinical and economic outcomes, potentially limiting our inclusion of studies that purely focused on either independently. For example, researchers have estimated other indirect costs attributable to LD such as “spending less time outdoors” and “less outdoor recreation” potentially leading to a substantial welfare loss each year [42].

## Recommendations for future economic evaluations of LD

To align with best practices frequently cited by health economists, we recommend LD researchers consider the following [43]:

- *Report both health sector and societal perspectives* – patients with LD experience significant costs outside of the formal health system (e.g. absenteeism, presenteeism, time, care not covered by health insurance) that should be captured in a societal perspective model.
- *Perform multiple sensitivity analyses on key assumptions* – as with any economic model, uncertainty analyses help test the main assumptions. Misdiagnoses, costs outside traditional health systems, patient heterogeneity, and provider heterogeneity may significantly impact the results of any cost-effectiveness or budget impact model in LD but may be difficult to ascertain. Expert opinion may be the best source for some of these variables and it will be important to test many of these assumptions independently.
- *Engage LD patients throughout the study* – by working directly with LD patients as advisors during economic model development, health economists can gain valuable perspectives that may not be captured in the published literature.

## Conclusions

Evaluations of clinical and economic outcomes of LD are primarily economic models or observational analyses and focus on formal health care. Less evidence exists for the broader impact of informal care or costs outside of the health system frequently experienced by LD patients.

## Supplementary information

**Additional file 1: Table S1.** PRISMA checklist.

## Data Availability

All data generated during this study are included in this published article.

## Abbreviations

LD: Lyme disease
PTLDS: post-treatment Lyme disease syndrome
PRISMA: preferred reporting items for systematic reviews and meta-analyses

## References

[1] Centers for Disease Control and Prevention. Lyme disease. Centers for Disease Control and Prevention; 2019. https://www.cdc.gov/lyme/index.html. Accessed 13 Jan 2019.

[2] Wormser GP, Dattwyler RJ, Shapiro ED, Halperin JJ, Steere AC, Klempner MS (2006). The clinical assessment, treatment, and prevention of Lyme disease, human granulocytic anaplasmosis, and babesiosis: clinical practice guidelines by the Infectious Diseases Society of America. Clin Infect Dis..

[3] Warshafsky S, Lee DH, Francois LK, Nowakowski J, Nadelman RB, Wormser GP (2010). Efficacy of antibiotic prophylaxis for the prevention of Lyme disease: an updated systematic review and meta-analysis. J Antimicrob Chemother..

[4] Sanchez E, Vannier E, Wormser GP, Hu LT (2016). Diagnosis, treatment, and prevention of Lyme disease, human granulocytic anaplasmosis, and babesiosis: a review. JAMA..

[5] Aucott JN, Crowder LA, Kortte KB (2013). Development of a foundation for a case definition of post-treatment Lyme disease syndrome. Int J Infect Dis..

[6] Ali A, Vitulano L, Lee R, Weiss TR, Colson ER (2014). Experiences of patients identifying with chronic Lyme disease in the healthcare system: a qualitative study. BMC Fam Pract..

[7] Onukwugha E, McRae J, Kravetz A, Varga S, Khairnar R, Mullins CD (2015). Cost-of-illness studies: an updated review of current methods. Pharmacoeconomics..

[8] Larg A, Moss JR (2011). Cost-of-illness studies: a guide to critical evaluation. Pharmacoeconomics..

[9] Mattingly TJ, Mullins CD, Onukwugha E (2016). Publication of cost-of-illness studies: does methodological complexity matter?. Pharmacoeconomics..

[10] Rice DP (2000). Cost of illness studies: what is good about them?. Inj Prev..

[11] Bloom BS, Bruno DJ, Maman DY, Jayadevappa R (2001). Usefulness of US cost-of-illness studies in healthcare decision making. Pharmacoeconomics..

[12] Šmit R, Postma MJ (2015). Lyme borreliosis: reviewing potential vaccines, clinical aspects and health economics. Expert Rev Vaccines..

[13] Mac S, da Silva SR, Sander B (2019). The economic burden of Lyme disease and the cost-effectiveness of Lyme disease interventions: a scoping review. PLoS ONE..

[14] Moher D, Liberati A, Tetzlaff J, Altman DG, PRISMA Group (2009). Preferred reporting items for systematic reviews and meta-analyses: the PRISMA statement. PLoS Med..

[15] Sanders GD, Neumann PJ, Basu A, Brock DW, Feeny D, Krahn M (2016). Recommendations for conduct, methodological practices, and reporting of cost-effectiveness analyses. JAMA..

[16] Berende A, Nieuwenhuis L, Ter Hofstede HJM, Vos FJ, Vogelaar ML, Tromp M (2018). Cost-effectiveness of longer-term *versus* shorter-term provision of antibiotics in patients with persistent symptoms attributed to Lyme disease. PLoS ONE..

[17] Johnson L, Wilcox S, Mankoff J, Stricker RB (2014). Severity of chronic Lyme disease compared to other chronic conditions: a quality of life survey. PeerJ..

[18] Joss AWL, Davidson MM, Ho-Yen DO, Ludbrook A (2003). Lyme disease—what is the cost for Scotland?. Public Health..

[19] Lantos PM, Brinkerhoff RJ, Wormser GP, Clemen R (2013). Empiric antibiotic treatment of Erythema migrans-like skin lesions as a function of geography: a clinical and cost effectiveness modeling study. Vector Borne Zoonotic Dis..

[20] Lightfoot RW, Luft BJ, Rahn DW, Steere AC, Sigal LH, Zoschke DC (1993). Empiric parenteral antibiotic treatment of patients with fibromyalgia and fatigue and a positive serologic result for Lyme disease: a cost-effectiveness analysis. Ann Intern Med..

[21] Lohr B, Müller I, Mai M, Norris DE, Schöffski O, Hunfeld K-P (2015). Epidemiology and cost of hospital care for Lyme borreliosis in Germany: lessons from a health care utilization database analysis. Ticks Tick Borne Dis..

[22] Magid D, Schwartz B, Craft J, Schwarts JS (1992). Prevention of Lyme disease after tick bites: a cost-effectiveness analysis. N Engl J Med..

[23] Meltzer M, Dennis D, Orloski K (1999). The cost effectiveness of vaccinating against Lyme disease. Emerg Infect Dis..

[24] Nichol G, Dennis DT, Steere AC, Lightfoot R, Wells G, Shea B (1998). Test-treatment strategies for patients suspected of having Lyme disease: a cost-effectiveness analysis. Ann Intern Med..

[25] Shadick NA, Liang MH, Phillips CB, Fossel K, Kuntz KM (2001). The cost-effectiveness of vaccination against Lyme disease. Arch Intern Med..

[26] van den Wijngaard CC, Hofhuis A, Wong A, Harms MG, de Wit GA, Lugnér AK (2017). The cost of Lyme borreliosis. Eur J Public Health..

[27] Boudreau CR, Lloyd VK, Gould ON (2017). Motivations and experiences of Canadians seeking treatment for Lyme disease outside of the conventional Canadian health-care system. J Patient Exp..

[28] Zhang X, Meltzer MI, Peña CA, Hopkins AB, Wroth L, Fix AD (2006). Economic impact of Lyme disease. Emerg Infect Dis..

[29] Maes E, Lecomte P, Ray N (1998). A cost-of-illness study of Lyme disease in the United States. Clin Ther..

[30] Drew D, Hewitt H (2006). A qualitative approach to understanding patients’ diagnosis of Lyme disease. Public Health Nurs..

[31] Eckman MH, Steere AC, Kalish RA, Pauker SG (1997). Cost effectiveness of oral as compared with intravenous antibiotic therapy for patients with early Lyme disease or Lyme arthritis. N Engl J Med..

[32] Fix AD, Strickland GT, Grant J (1998). Tick bites and Lyme disease in an endemic: setting problematic use of serologic testing and prophylactic antibiotic therapy. J Am Med Assoc..

[33] Gasmi S, Ogden NH, Leighton PA, Adam-Poupart A, Milord F, Lindsay LR (2017). Practices of Lyme disease diagnosis and treatment by general practitioners in Quebec, 2008–2015. BMC Fam Pract..

[34] Henningsson AJ, Malmvall BE, Ernerudh J, Matussek A, Forsberg P (2010). Neuroborreliosis—an epidemiological, clinical and healthcare cost study from an endemic area in the south-east of Sweden. Clin Microbiol Infect..

[35] Hsia EC, Chung JB, Schwartz JS, Albert DA (2002). Cost-effectiveness analysis of the Lyme disease vaccine. Arthritis Rheum..

[36] Johnson L, Aylward A, Stricker RB (2011). Healthcare access and burden of care for patients with Lyme disease: a large United States survey. Health Policy..

[37] Lakdawalla DN, Doshi JA, Garrison LP, Phelps CE, Basu A, Danzon PM (2018). Defining elements of value in health care—a health economics approach: an ISPOR Special Task Force Report [3]. Value Health.

[38] Adrion ER, Aucott J, Lemke KW, Weiner JP (2015). Health care costs, utilization and patterns of care following Lyme disease. PLoS ONE..

[39] Brouwer WBF (2018). The inclusion of spillover effects in economic evaluations: not an optional extra. Pharmacoeconomics..

[40] Lin P-J, D’Cruz B, Leech AA, Neumann PJ, Sanon Aigbogun M, Oberdhan D (2019). Family and caregiver spillover effects in cost-utility analyses of Alzheimer’s disease interventions. Pharmacoeconomics..

[41] Slunge D (2015). The willingness to pay for vaccination against tick-borne encephalitis and implications for public health policy: evidence from Sweden. PLoS ONE..

[42] Berry K, Bayham J, Meyer SR, Fenichel EP (2018). The allocation of time and risk of Lyme: a case of ecosystem service income and substitution effects. Environ Resour Econ..

[43] Neumann PJ, Sanders GD, Russell LB, Siegel JE, Ganiats TG (2017). Cost-effectiveness in health and medicine.

